# Effects of the Inclusion of Different Levels of Dietary Sunflower Hulls on the Colostrum Compositions of Ewes

**DOI:** 10.3390/ani11030777

**Published:** 2021-03-11

**Authors:** Mohsen M. Alobre, Mutassim M. Abdelrahman, Ibrahim A. Alhidary, Abdulrahman S. Alharthi, Riyadh S. Aljumaah

**Affiliations:** 1 Department of Animal Production, Faculty of Food and Agriculture, King Saud University, Riyadh 11451, Saudi Arabia; malobre@ksu.edu.sa (M.M.A.); ialhidary@ksu.edu.sa (I.A.A.); abalharthi@ksu.edu.sa (A.S.A.); rjumaah@ksu.edu.sa (R.S.A.); 2 Research and Extension Authority, Dhamar P. O. Box 87148, Yemen

**Keywords:** Naemi ewes, sunflower hulls, colostrum, saturated fatty acid, unsaturated fatty acids, lignin

## Abstract

**Simple Summary:**

Increasing the cost of roughages, as a result of high demand by livestock producers, leads to a search for alternative sources. Plant by-products such as sunflower hulls can be used as an alternative source of fiber. This manuscript is a part of ongoing research to identify the best levels to be used without negative effects on ruminant animals’ performances.

**Abstract:**

This study investigated the effects of supplementing different levels of sunflower hulls (SFH) to the complete feed of late-gestation pregnant ewes on the nutritive and fatty acids profile of colostrum at three and 48 h postpartum. In this study, 24 primiparous lactating Naemi ewes were randomly selected at parturition from four dietary groups as follows: (1) C (control), total mixed ration (TMR; 0% SFH), (2) S12, TMR1 with 12% SFH (level 1), (3) S20, TMR2 with 20% SFH (level 2), and (4) S28, TMR3 with 28% SFH (level 3). The body condition scores were estimated 30 days prepartum. Colostrum samples were collected at three and 48 h postpartum to measure the nutritive values and fatty acids profiles. Data were analyzed as a complete randomized design with repeated measures and via Pearson’s correlation and regression analyses. The results indicated a numerical correlation (R^2^ = 0.42; *p* < 0.09) between the body condition score and both colostrum fat and total solids. SFH increased the colostrum fat % (*p* < 0.05), especially for S12 and S20, following a cubic trend. Additionally, the colostrum from S12 and S20 ewes had a higher proportion of unsaturated fatty acids (USFAs), omega3 and lower levels of saturated fatty acids (SFAs), other than margaric acid (C17) and stearic acid (C18), SFA/USFA, and omega 6/omega 3. Furthermore, the regression analysis of the fatty acid classes and indices indicated a quadratic relationship between the parameters and SFH levels. The results confirm that the inclusion of SFH at levels greater than 20% may have a negative effect on some fatty acid parameters as a result of the high lignin intake. In conclusion, up to 20% SFH can be used in a complete feed for pregnant ewes without negative effects on the colostrum fat content and fatty acids profiles.

## 1. Introduction

For ruminants, the provision of complete feed formulation as a total mixed ration (TMR), mainly depends on the levels of dietary fiber, acid detergent fiber (ADF) and neutral detergent fiber (NDF); particularly, NDF levels influence the ruminal fermentation process, and many metabolic disorders can arise if the requirements are not met [1]. Late gestation is the most critical period for both ewes and unborn lambs. Maternal undernutrition, especially NDF, affects the colostrum and milk production and its nutritive value, which is associated with the survival and growth of newborns [2]. The quantity and source of fiber mainly affect the levels of volatile fatty acids (VFAs; acetic, propionic, and butyric acids) in the rumen, which, in turn, affect the pregnant ewes’ performance, colostrum, and milk fat content [3]. Recently, sunflower hulls (SFH) have begun to be considered to be an alternative to alfalfa hay, as well as other highly available cheap sources of fiber. Due to its high lignin content, SFH may have a negative effect on ruminant animals when included as the main source of dietary NDF [4]. Nonetheless, SFH can be included in complete feed as a partial source of NDF to reduce feed costs [5].

Body condition scoring is a useful management tool to evaluate the nutritional status and, consequently, the milk and colostrum nutritive values of pregnant ewes [6,7]. Several studies have examined the relationship between ewes’ body condition scores (BCS) and milk composition. Furthermore, body condition scoring is a means to estimate tissue mobilization as a main source of fat and energy for productivity [8]. Although with inconsistent results, other studies have found an association between BCS and milk yield and composition.

As in all mammals, colostrum and milk are the main source of food for lambs during the first weeks of life. Colostrum is a nutrient-rich fluid secreted by the mammary glands of female mammals after giving birth and during the first 24–48 h after birth; after which time, the secretion changes into milk [9]. In the rumen, the proportion of different VFAs affects the fat content and fatty acids profile of milk [10]. Acetic and butyric acids are the main precursors of milk fat, and propionic acid is the main precursor for glucose and, consequently, milk and colostrum lactose [11]. In colostrum, fats and fatty acids are the main dietary components that fuel the metabolic process [12] and health of newborns [13]. Unsaturated fatty acids (USFAs), such as linoleic and linolenic acids, play a critical role in the metabolic functions and general performance of newborns [14].

Given that previous studies are limited, the use of a byproduct as a source of NDF, such as SFH, must be investigated to specify the proper levels to be used in the complete feed without any negative effect on the ewes’ BCS, colostrum fat, and fatty acids. As such, this study investigated the effect of including different levels of SFH to the complete feed for late-gestation ewes on colostrum nutritive values and fatty acids profile at three and 48 h postpartum.

## 2. Materials and Methods

### 2.1. Experimental Design and Diets

This study was conducted at the Experimental Station of Department of Animal Production, College of Food and Agriculture Sciences, King Saud University, Riyadh. All research was performed according to the Scientific Research Ethics Committee at King Saud University (approval number: KSU-SE-20-27).

The trial started by using 84 primiparous pregnant ewes (60 prepartum) fed 4 different diets containing different levels of SFH. The dietary groups were as follows: C (control), TMR with 0% SFH, S12: TMR with 12% SFH (level 1), S20: TMR with 20% SFH (level 2), and S28: TMR with 28% SFH (level 3). Three replicates were randomly allocated to one of the four dietary treatments. All ewes were offered their assigned diet formulated to meet all nutritional requirements according to the National Research Council (NRC, 2007), as shown in Table 1. At parturition, 24 Naemi ewes were randomly selected from the 84 pregnant ewes at parturition, 6 ewes from each dietary group, for colostrum sampling and other measurements. Selected ewes were fed as a group in shaded separate pens (7.0 m long × 6.0 m wide; 2 ewes per pen). Each pen was equipped with a feed trough and a water bucket. All required vaccines and medical treatments were performed according to standard practices.

### 2.2. Body Condition Scores

For each pregnant ewe, BCS was assessed at minus 30 days prepartum using the dorsal palpation technique [15]. BCS measurements are based on height, weight, and age and follow an interval range of 0.5 points. (2, 2.5, 3, 3.5, and >4) for each treatment: C, S12, S20, and S28.

### 2.3. Colostrum Sampling

Colostrum samples (60 mL) were collected via hand milking from each ewe, 6 ewes per treatment, during suckling at 3 and 48 h postpartum. After the collection, a 10-mL colostrum subsample from each ewes were taken by thoroughly mixed colostrum and stored at −20°C for further analysis of the nutrient composition (i.e., protein, fat, lactose, total solid, and FA profile). The fat, protein, lactose, and total soluble solids percentages of colostrum were determined using Milko-Scan (Minor Type 78100, Foss Electric, DK-3400 Hilleroed/Denmark).

### 2.4. Fatty Acids Profile

Colostrum samples were extracted using the procedures described by Luna et al. 2005 [16] to determine their FA profiles. Samples were centrifuged at 5000 rpm for 10 min at 4 °C, followed by floating globule collection. Triacylglycerols present in the colostrum fat were saponified and converted to fatty acid methyl esters (FAMEs) using the methods described by [17]. FAMEs were obtained by adding 2-mL hexane and 200 μL of 2-M sodium methoxide to 40-mg fat fraction. The fat mixture was heated in a water bath at 50 °C for a few seconds, and then, 200 μL of 2-M HCl was added. A 1-μL aliquot of the top layer was used to determine the fatty acid level of colostrum (MFAS). MFAS were identified and quantified using a gas chromatography-mass spectrometry ultra-instrument (GCMS-QP2010, Shimadzu, Kyoto, Japan) and an Rtx-1 column (30 m × 0.25 mm. 0.25-μm film thickness), with helium as the carrier gas at a flow rate of 1.41 mL min^−1^. The oven temperature was increased from 150 °C to 180 °C at a rate of 15 °C min^−1^, followed by an increase to 210 °C at 1 °C min^−1^. The temperatures of the injector and detector were 220 °C and 275 °C, respectively. For gas chromatography-mass spectrometry detection, an electron ionization system with ionization energy of 70 eV was used. The relative percentages of the individual FA were calculated based on the ratios of the peak areas of the fatty acids to the total peak area of all the fatty acids in the colostrum fat sample.

### 2.5. Statistical Analysis

Pearson’s correlation coefficient was calculated to evaluate the relationship between BCS and colostrum fat and total solids (TS) using the following model:

(1) $$R\;=\;\frac{\mathrm{Sxy}}{\mathrm{SX}\;\mathrm{SY}}$$

where Sx is the variance of x, Sy is the variance of y, and Sxy is the covariance between x and y.

The colostrum composition and fatty acids profile were analyzed as a complete randomized design with repeated measures using the PRO Mixed Model of SAS 2003, [18]. Dietary treatment (SFH level), time (3 and 48 h) of sampling, and the interaction between treatment and time were tested. The model for the performance of ewes and newborns was as follows:

yijk = μ + τi + δij + tk + (τ multiple t)ik + εijk i = 1, …, a; j = 1, …, b; k = 1, …, n(2)

where yijk is the observation ijk, μ is the overall mean, τi is the treatment effect, tk is the effect of time, (τ multiple t) is the effect of the interaction between treatment i and time k, δij is the random error with a mean of 0 and variance σ2 δ, the variance between animals (subjects) within the treatment, and it is equal to the covariance between the repeated measurements within animals, and εijk is the random error with a mean of 0 and variance σ2, the variance between measurements within animals.

The coefficient of determination (R^2^) and *t*-test *p*-values for regression parameters were used as criteria for selecting the best fitting model:

yijk = μ + τi + δij + tk + (τ multiple t)ik + εijk i = 1, (3)

yi = β0 + β1 × i1 + β 2 × i2 + … β pxip + εij for i = 1,2, …, n(4)

where yi is the dependent variable (colostrum composition); X and Xi are the independent variables (0%, 12%, 20%, and 28% SFH); βo, β2, …, β3 are the regression parameters; and εij is the random error.

## 3. Results

### 3.1. Body Condition Scores and Colostrum Composition

There was no significant effect of treatment (*p* > 0.05) on the BCS of ewes at −30 days prepartum; the average BCS scores were 3.40, 3.31, 3.00, and 3.40 for ewes from the C, S12, S20, and S28 treatments, respectively. Numerically, S20 ewes had the lowest mean BCS.

**Figure 1:**
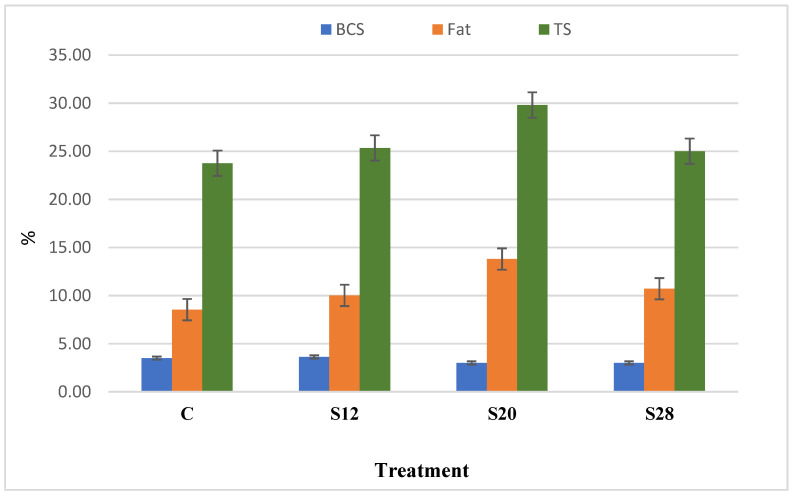
The relationship between the body condition score, colostrum fat, and colostrum total solid (TS) of ewes fed a complete diet with different levels of sunflower hulls.

Figure 1 demonstrates the relationship between the ewes’ BCS and their colostrum composition. Although with no other aspects, results indicated a correlation between BCS and both colostrum fat and TS. There was a numerical, but nonsignificant, correlation (R^2^ = 0.42; *p* < 0.09) between the BCS and fat composition for all groups. A high correlation was detected between the fat and TS content (R^2^ = 0.77; *p* < 0.003). For C, S12, S20, and S28 ewes, the fat values were 8.54%, 10.02%, 13.8%, and 10.72%, and the TS values were 23.77%, 25.35%, 29.81%, and 25.01%, respectively.

Table 2 shows the effect of the SFH level on the colostrum nutritive values at three and 48 h postpartum. SFH supplementation had a significant effect (*p* < 0.05) on the colostrum fat, lactose, and TS. Similar trends were reported for the effect of sampling time on all variables. Moreover, there was no significant interaction effect on all the variables.

**Figure 2:**
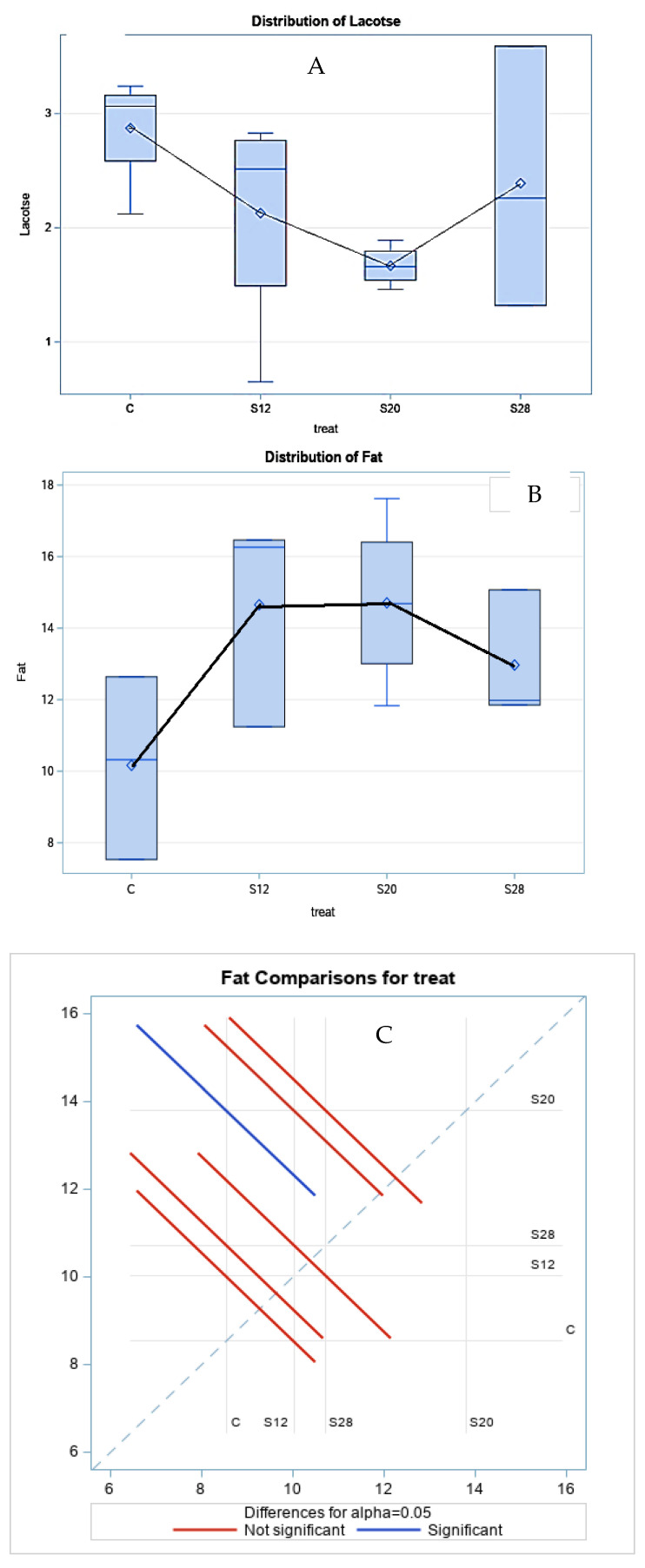
The effect of different levels of sunflower hulls on the colostrum composition at 3 h postpartum, as assessed via the regression analysis (**A**), Lactose distribution; (**B**), Fat distribution; (**C**) Fat comparisons for treatment.

Table 3 demonstrates the effect of different levels of SFH on the colostrum composition at three hours postpartum. Fat concentrations were significantly higher (*p* < 0.05) in the colostrum from S12, S20, and S28 ewes compared with those from other groups. A similar trend was noted for lactose, with lower values for ewes from S12 and S20 ewes. The treatment had a cubic effect (*p* < 0.05) on the fat and lactose percentages, as shown in Figure 2, which shows the regression trend. There was no significant effect of SFH on the protein or TS content.

**Figure 3:**
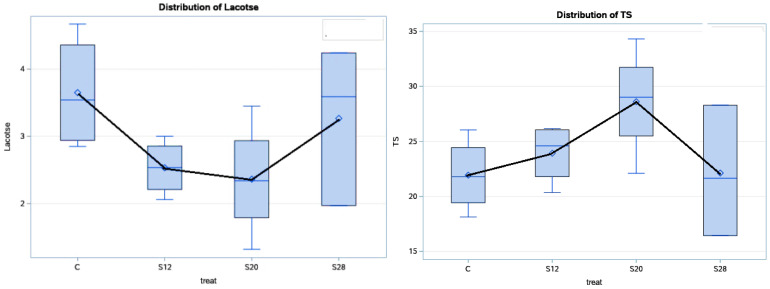
The effect of dietary treatment on the colostrum total solid and lactose contents at 48 h.

Table 4 and Figure 3 show the effects of using different levels of SFH on the colostrum composition at 48 h postpartum. Treatment affected the lactose and TS concentration (*p* < 0.05) in the colostrum from S12 and S20 ewes, compared with the other groups, with higher values for TS and lower values for lactose. Moreover, the effect of the treatment had a cubic effect (*p* < 0.05) on the lactose and TS percentages. The fat and protein contents were not significantly affected (*p* > 0.05) by the treatment, although their values were numerically higher in the colostrum from S12 and S20 ewes compared with those from C and S28 ewes.

**Figure 4:**
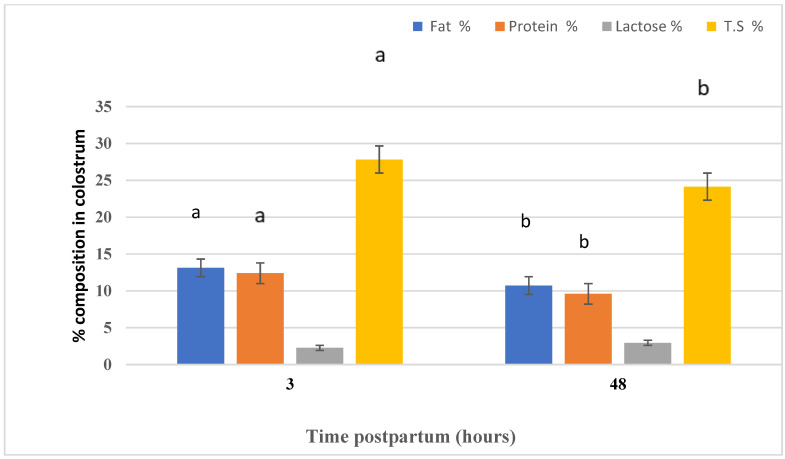
The effect of the differences in time of sampling on the colostrum composition.

Figure 4 highlights the effect of time (three and 48 h postpartum) on the colostrum composition of Naemi ewes. Time postpartum had an effect on the colostrum fat, protein, and TS, whereas lactose did not differ between the two periods. Fat, lactose, and TS concentrations in the colostrum were higher (*p* < 0.05) in three h postpartum samples than in 48 h postpartum samples.

### 3.2. Colostrum Fatty Acids Profile

Table 5 shows the effect of the treatment on the SFA and USFA profiles (g/100 g fat) of the colostrum. In terms of the fatty acid profiles, different dietary concentrations of SFH (S12, S20, and S28) significantly affected the proportions of C6 (*p* < 0.01), C8 (*p* < 0.05), C10 (*p* < 0.001), C12 (*p* < 0.001), C14 (*p* < 0.05), and C15 (*p* < 0.05), compared with the control group. The lowest value for C12 was noted in the colostrum from ewes fed 12% SFH. C6:0, C8:0, and C18:0 were significantly higher (*p* < 0.05) in the colostrum from S28 ewes compared with those from the other groups. The colostrum from ewes belonging to the S28 group had the highest levels of C6 and C8 compared with all the other groups. Additionally, the proportions of C10 and C12 were significantly lower (*p* < 0.001) in the colostrum from the S12 ewes. The proportions of C17 and C18 were affected by the SFH supplementation, leading to higher values in SFH-supplemented ewes compared with those noted in ewes in the control group. Conversely, significantly lower values of C19 were noted in the colostrum from S20 ewes compared with the control ewes, but these levels did not differ compared with the S12 and S28 ewes.

Data related to USFA levels in ewes’ colostrum showed a varied response to SFH supplementation compared with the control. Ewes fed S28 showed significantly lower (*p* < 0.05) values for C18:1 ∆ 9c, C18:1 ∆ 11t, and C18:2 9c 11t CLA but higher values for C18:2 9.12 t, C18:2 6.9.12 c, C18:2∆ 9 c.11, and C22:5.7 10.13.16.19. For S12, significantly higher (*p* < 0.05) values were reported for C18:1 ∆ 9c compared with S28. Furthermore, ewes fed S20 showed higher values for C18:1 ∆ 11t and C18:2 9c 11t CLA. 

Conversely, there was no significant effect of time postpartum nor the interaction between time postpartum and treatment on all fatty acids profiles, except for C18:0, which was affected by the time postpartum.

**Figure 5:**
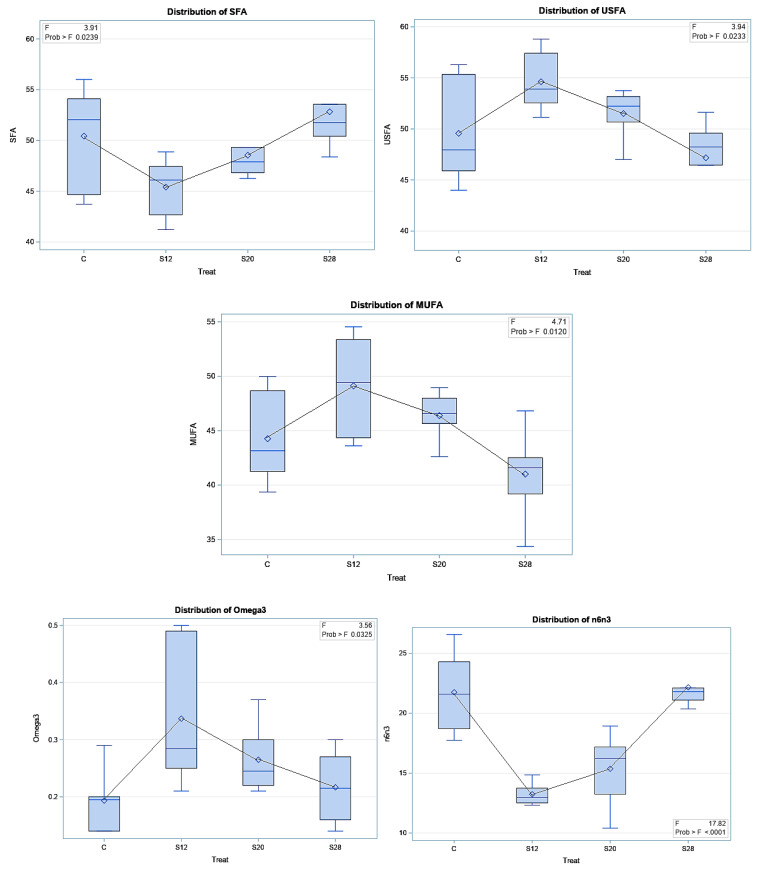
The effect of different levels of sunflower hulls on colostrum fatty acid indices (i.e., saturated fatty acids, SFA; unsaturated fatty acids, USFA; monounsaturated fatty acids, MUFA; omega3; and SFA/USFA, saturated fatty acids per unsaturated fatty acids; omega6/omega3, n6/n3), as assessed via the regression analysis.

Table 6 demonstrates the effect of SFH supplementation on colostrum FA classes and indices. SFH supplementation led to significant differences for SFA (*p* < 0.05), USFA (*p* < 0.05), MUFA (*p* < 0.01), omega3 (*p* < 0.05), SFA/USFA (*p* < 0.05), and omega6/omega3 (*p* < 0.001). Significantly lower SFA levels (*p* < 0.05) were found in the colostrum from S12 ewes compared with those from C and S28 ewes. A higher proportion of USFA was found in the colostrum from S12 and S20 ewes compared with those from control and S28 ewes, whereas these ewes had lower values for SFA/USFA and omega6/omega3. Furthermore, the results of the regression analysis of the FA classes and indices are reported in Figure 5. A quadratic trend was found for SFA (*p* < 0.01), USFA (*p* < 0.01), MUFA (*p* < 0.01), omega3 (*p* < 0.01), SFA/USFA (*p* < 0.001), and omega 6/omega 3 (*p* < 0.001).

## 4. Discussion

Late gestation is the most critical period for ewes and their unborn lambs. Maternal undernutrition affects colostrum and milk production and its nutritive value, which is associated with the survival and growth of newborns [19]. The use of byproducts, such as SFH as an alternative source of NDF, has been recently introduced into ruminant diets, but the proper inclusion levels, especially for the late-gestation period, have not been identified. The ruminal contents of VFAs have been reported to be highly correlated with ewe BCS, colostrum and milk nutritive values, long-chain FAs, and consequently, newborn mortality rate and health [20,21].

The BCSs of ewes during late gestation (−30 days prepartum) were between 3 and 3.4 and were not affected by SFH levels. Conversely, the BCS for all groups showed a numerical correlation with colostrum fat and TS; this suggests that the accumulation of fat in the ewes’ body tissues during late gestation, up to a BCS of 3.4, affects the fat content of their colostrum. This finding is in agreement with the results of Hull et al. [22], who reported that BCS affected the milk yield, milk fat, and ash content in crossbred Holstein Friesian dairy cows, with a positive relationship between BCS and milk fat, protein, lactose, and TS. Murat and Hakan [23] found a significant effect of low BCS (1.5–2.5) on milk fat synthesis in the mammary gland. Similar results were found by Zahraddeen et al. [24], who reported a significant influence of ewes’ BCS on milk composition parameters such as TS, lactose, crude protein, fat contents, pH, and ash. This suggests that ewe BCS is crucial to maintaining colostrum nutritive values, especially fat and TS. According to our results, feeding pregnant ewes up to 20% SFH enhances the fat content in their colostrum, leading to improving their newborns’ health.

In this study, the fiber (NDF), lignin, and FA profile of the diets played a crucial rule and may have affected the colostrum nutritive values. The NDF levels of C, S12, S20, and S28 were 37.59%, 38.74%, 36.50%, and 41.52%, respectively, whereas the lignin levels were 7.37%, 6.99%, 8.88%, and 9.07%, respectively, and fiber levels were 18.26%, 20.78%, 22.18%, and 21.81%, respectively. Moreover, the FA profile of the diets was reported in Table 1. Diets supplemented with SFH had higher levels of fiber NDF and lignin; lignin is the most limiting factor, since it negatively affects the digestion of fibre. As such, grinding and pelleting the feed, as well as reducing the particle size, may reduce the negative effects of lignin on fiber fermentation in the rumen.

SFH supplementation led to a significant difference in colostrum fat and lactose percentages; S12, S20, and S28 ewes had higher colostrum fat compared with the control ewes, whereas S12 and S20 ewes had lower colostrum lactose compared with control and S28 ewes. Fat percentage is mainly affected by the NDF content of the diet because of its involvement in the production of acetic acid in the rumen by microorganisms, increasing the ratio of acetic:propionic acid [25]. The higher levels of NDF in the S12, S20, and S28 diets, compared with those in the control, led to an increase in colostrum fat, although the colostrum fat percentage was lower for S28 ewes compared with those for S12 and S20 ewes. The reasons for this finding may be caused by a high lignin 9.07 intake from S28 diet, which may negatively affect the rumen fermentation process of NDF. This lactose result is expected because of an increase in the fat percentage due to decreased propionic acid production in the rumen. Our results are in line with those of Capper et al. [26], who observed that fiber levels during late pregnancy improved the colostrum quality.

Our results for protein, fats, lactose, and nonfat solids correspond to the normal range for Naemi ewes, as reported by Ahmadi [27] and Peka et al. [28]. The time of sampling had a significant effect on fat, lactose, and TS but had no effect on the protein. Similar results were reported by Bernabucci et al. [29], who found that the chemical composition of ewes’ colostrum had different fat and protein contents in the first hours after lambing. Banchero et al. [30] found that the fat and protein content of colostrum did not differ in single- and twin-bearing ewes at parturition and 10 h after birth, but the colostrum from single-bearing ewes had a higher content of lactose than twin-bearing ewes at three and six hours postpartum. Thus, the findings from different authors are not in agreement, which may be due to any other number of factors.

There is no existing data on the effects of SFH on colostrum quality, especially the FA profile. Colostrum FA biosynthesis is a complex process that involves many organs, metabolic pathways, and the functioning of the mammary gland [31,32]. Fatty acid profile analyses showed that ewes’ colostrum was rich in saturated acids, as was previously reported by Murat and Hakan [33]. In our results, the interaction between dietary SFH levels and lactation time did not significantly affect the colostrum FA profiles. As a general trend, colostrum from ewes fed the S12, S20, and S28 diets had a higher proportion of most USFA compared with those fed the control diet. By contrast, there was no significant effect of time nor of the interaction between time and treatment on most FA profiles; the only exception was C18:0, which was affected by the sampling time postpartum. The S12 and S20 treatments, with lower proportions of short- and medium-chain fatty acids, may have reduced the de novo synthesis of FA in the mammary gland. Colostrum from ewes fed the S12 and S20 diets had a reduced percentage of SFA. β-Hydroxybutyrate and acetate, as a result of fiber fermentation in the rumen, are the major sources for de novo FA synthesis [34]. Conversely, C17:0 and C18:0, which are long-chain FA, increased with supplementation; these were higher in the colostrum from S12, S20, and S28 ewes compared with those from C ewes. These long-chain fatty acids mainly originate from circulating blood lipids after absorption from the small intestine or mobilization from adipose tissue.

SFH supplementation led to significant differences in the colostrum SFA (*p* < 0.05), USFA (*p* < 0.05), MUFA (*p* < 0.01), omega3 (*p* < 0.05), SFA/USFA (*p* < 0.05), and omega6/omega3 (*p* < 0.001). SFA levels were lower in the colostrum from S12 ewes compared with colostrum from C and S28 ewes. The colostrum proportion of USFA was higher, whereas SFA/USFA and omega6/omega3 were lower in the colostrum from the S12 and S20 ewes compared with those from C and S28 ewes. Furthermore, SFA (*p* < 0.01), USFA (*p* < 0.01), MUFA (*p* < 0.01), omega3 (*p* < 0.01), SFA/USFA (*p* < 0.001), and omega6/omega3 (*p* < 0.001) followed a quadratic trend. Payne et al. [35] reported that short-chain fatty acids are mainly a result of acetic acid in the rumen, whereas long-chain fatty acids are a result of dietary fat and tissue mobilization. As such, our colostrum FA profile results are likely linked to dietary FAs levels, particularly given the high levels of long-chain fatty acids.

Hur et al. [36] reported that linoleic acid (C18:2) is released from dietary lipids as a result of bacterial lipase activity in the ruminant digestive tract, which further undergoes biohydrogenation and is later absorbed into the small intestine. This leads to the formation of C18:0, which is an important substrate for oleic acid (C18:1 n-9c) production, as was shown in ewes fed S12, S20, and S28, respectively, from the USFA synthesis (Table 6). Bernard et al. [37] found that, in milk fat, fatty acids shorter than C16:0 are produced by de novo fatty acid synthesis in the mammary gland. High levels of linoleic acid can also contribute to an increase in the concentration of vaccenic acid (C18:1 n-11t) in the rumen [38]. Moreover, excess linoleic is transported to the mammary gland, where it is subsequently converted to conjugated linoleic acid (c-9, t-11; [39]). In this study, the high fiber and NDF levels in the feed supplemented with SFH may have limited the NDF microbial fermentation process in the rumen because of high lignin levels, especially in S28 ewes. As such, a higher concentration of C17:0 was found in SFH-supplemented groups (1.09, 1.07, and 1.05 g/100 g of the total FA for S12, S20, and S28, respectively) compared with the control group (0.45 g/100 g). Our results are in line with the findings by Weimer et al. [40], who reported that the ruminal cellulolytic bacterial population increased with a higher dietary NDF, which may affect the ruminal fermentation process and, consequently, its end products.

## 5. Conclusions

The addition of SFH to TMR, as source of NDF for pregnant Naemi ewes, led to maintain ewes’ BCS from 3 to 3.4, which were numerically correlated to colostrum fat and total solids. Complete feed with 12% and 20% SFH improved the nutritive value of colostrum (i.e., fat, lactose, and TS) and led to higher proportions of USFA and omega3 and lower proportions of SFA, with the exception of C17 and C18, SFA/USFA, and omega 6/omega 3. Furthermore, the regression analysis of the FA classes and indices revealed a quadratic relationship between the parameters and SFH levels.

## Figures and Tables

**Table 1:** Experimental diet composition and fatty acid (FA) profiles.

Treatments	C	S12	S20	S28
**Chemical Composition (%)**				
Dry matter	90.39	88.47	88.74	88.54
Protein	14.86	14.55	14.18	14.98
Fiber	18.26	20.78	22.16	21.81
Ash	14.25	6.61	6.34	5.88
Fat	4.02	4.35	4.35	4
GE (Kcal/Kg)	3641	3613	3710	3744
ADF	28.46	30.25	30.66	29.55
NDF	37.59	38.74	39.5	41.52
Lignin	7.37	6.99	8.88	9.07
**Fatty acid profile** **(g/100 g total FA)**				
C6:0	0.19	0.1	0.09	0.13
C8:0	1.2	1.26	1.62	1.5
C10:0	1.37	1.49	1.52	1
C12:0	22.03	24.92	21.76	15.41
C14:0	8.53	8.88	7.8	5.42
C15:0	0.13	0.15	0.09	0.09
C16:0	16.27	14.04	11.43	14.88
C17:0	0.04	0.07	0.01	0.08
C18:0	4.32	3.87	2.99	3.47
C18:1 ∆ 9t	0.15	0	0.05	0.12
C18:1 ∆ 9c	31.53	26.65	25.72	33.42
C18:2 9, 11cis; C18:2 10t 12	13.4	17.42	25.63	23.36
C18:3 cis 9, 12, and 15,	0.62	0.43	0.4	0.58
C20	0.21	0.71	0.89	0.52
SFA	54.3	55.49	48.2	42.51
MUFA	31.68	26.65	25.77	33.55
PUFA	14.02	17.85	26.03	23.94

C, 0% sunflower hulls (SFH); S12, 12% SFH; S20, 20% SFH; S28, 28% SFH; ∆, Delta SFA, saturated fatty acids; MUFA, monounsaturated fatty acid; and PUFA, polyunsaturated fatty acid.

**Table 2:** The effect of different levels of sunflower hulls on the colostrum composition (%).

Variable	Treatment				Statistical Significance			
	C	S12	S20	S28	SEM	Treatment	Time	Treatment * Time
Fat %	8.54	10.02	13.80	10.72	0.59	*	*	NS
Protein %	9.87	10.96	12.15	10.99	0.34	NS	*	NS
Lactose %	3.26	2.33	2.01	2.82	0.13	*	*	NS
TS %	23.77	25.35	29.81	25.01	0.65	*	*	NS

C, 0% sunflower hulls (SFH); S12, 12% SFH; S20, 20% SFH; S28, 28% SFH; TS, total solids; NS, no significant effect; * significant effect (*p* < 0.05); and SEM: standard error of the mean.

**Table 3:** The effect of different levels of sunflower hulls on the colostrum composition, as assessed 3 h postpartum.

Variable	Treatment				Statistical Significance				
	C	S12	S20	S28	SEM	R^2^	L	C	Q
Fat %	10.16 ^a^	14.65 ^b^	14.70 ^b^	12.96 ^b^	0.79	0.44	NS	*	NS
Protein %	12.01	11.99	13.43	12.14	0.35	0.22	NS	NS	NS
Lactose %	2.87 ^a^	2.12 ^b^	1.66 ^c^	2.39 ^a^	0.21	0.31	NS	*	NS
TS %	25.61	26.79	31.01	27.90	1.09	0.26	NS	NS	NS

C, 0% sunflower hulls (SFH); S12, 12% SFH; S20, 20% SFH; S28, 28% SFH; and TS, total solids. ^a,b,c^ Means within a column with different superscripts significantly differ. L: linear, C: cubic, and Q: quadratic. * Significant effect (*p* < 0.05) and NS, no significant effect.

**Table 4:** The effect of different levels of sunflower hulls on the colostrum composition, as assessed 48 h postpartum.

Variable	Treatment				Statistical Significance				
	C	S12	S20	S28	SEM	R^2^	L	C	Q
Fat %	9.60	10.66	14.12	8.47	0.86	0.32	NS	NS	NS
Protein %	7.72	9.94	10.86	9.85	0.66	0.23	NS	NS	NS
Lactose %	3.65 ^a^	2.53 ^b^	2.36 ^b^	3.26 ^a^	0.24	0.35	NS	*	NS
TS %	21.93 ^a^	23.92 ^b^	28.61 ^b^	22.12 ^a^	1.22	0.36	NS	*	NS

C, 0% sunflower hulls (SFH); S12, 12% SFH; S20, 20% SFH; S28, 28% SFH; and TS, total solids. ^a,b^ Means within a column with different superscript letters significantly differ. L: linear, Q: quadratic, and C: cubic. * Significant effect (*p* < 0.05) and NS, no significant effect.

**Table 5:** Fatty acids profile of colostrum samples from ewes fed different levels of sunflower hulls.

Component	C	S12	S20	S28	SEM	Treatment	Time	Treatment * Time
SFAs (g/100 g)								
C6:0	0.41 ^b^	0.43 ^b^	0.49 ^b^	0.72 ^a^	0.04	**	NS	NS
C8:0	0.45 ^ab^	0.30 ^b^	0.40 ^b^	0.86 ^a^	0.08	*	NS	NS
C10:0	1.46 ^a^	0.55 ^b^	1.19 ^a^	1.44 ^a^	0.09	***	NS	NS
C12:0	2.77 ^a^	0.67 ^c^	1.47 ^b^	1.48 ^b^	0.18	***	NS	NS
C14:0	8.49	5.57	6.43	6.52	0.53	NS	NS	NS
C15:0	0.17	0.10	0.11	0.08	0.01	NS	NS	NS
C16:0	27.02	25.15	25.27	26.34	0.65	NS	NS	NS
C17:0	0.45 ^b^	1.09 ^a^	1.07 ^a^	1.05 ^a^	0.09	*	NS	NS
C18:0	6.67 ^b^	9.75 ^a^	10.29 ^a^	12.79 ^c^	0.65	**	*	NS
C19:0	0.25 ^a^	0.19 ^ab^	0.13 ^b^	0.16 ^ab^	0.02	*	NS	NS
C20:0	0.13	0.15	0.65	0.13	0.12	NS	NS	NS
C22:0	0.04	0.02	0.02	0.32	0.03	NS	NS	NS
Remaining acids	2.12	1.43	1.02	0.93				
USFAs (g/100 g)								
C14:1 ∆ 9C	0.03	0.07	0.03	0.07	0.01	NS	NS	NS
C15. 9 Methyl C15	0.04	0.03	0.02	0.03	0.01	NS	NS	NS
C16: 1∆7C	0.31	0.32	0.24	0.28	0.02	NS	NS	NS
C16:1∆9C	2.04 ^a^	1.26 ^b^	1.34 ^b^	1.30 ^b^	0.10	*	NS	NS
C18:1 ∆ 9c	36.37 ^b^	43.36 ^b^	39.79 ^ab^	35.03 ^a^	1.06	*	NS	NS
C18:1 ∆ 11t	1.11 ^a^	1.06 ^ab^	1.12 ^a^	0.83 ^b^	0.04	*	NS	NS
C18: 1∆ 6t	0.25	0.24	0.30	0.19	0.02	NS	NS	NS
C18:2 9.12 t	0.16 ^b^	0.08 ^c^	0.08 ^c^	0.23 ^a^	0.02	**	NS	NS
C18:2∆ 9 c.11	3.09 ^a^	3.21 ^a^	2.94 ^a^	3.45 ^b^	0.14	*	NS	NS
C18:2 6.9.12 c	0.12 ^a^	0.16 ^b^	0.10 ^a^	0.17 ^b^	0.04	*	NS	NS
C18:3 cis 9.12.15	0.14 ^a^	0.10 ^b^	0.11 ^a^	0.18 ^b^	0.04	**	NS	NS
C18:2 9c 11t CLA	0.11 ^a^	0.16 ^b^	0.17 ^b^	0.10 ^a^	0.03	*	NS	NS
C22:5 7 10.13.16.19	0.12	0.10	0.23	0.65	0.11	NS	NS	NS
Remaining acids	5.67	4.46	5.11	4.59				

C, 0% sunflower hulls (SFH); S12, 12% SFH; S20, 20% SFH; S28, 28% SFH; SFAs, saturated fatty acids; USFAs, unsaturated fatty acids; NS, not statistically significant; * statistically significant at *p* < 0.05; and ** statistically significant at *p* < 0.01; *** statistically significant at *p* < 0.001. ^a,b,c^ Means within a row with different superscript letters significantly differ.

**Table 6:** Effect of sunflower hulls on colostrum fatty acid classes and indices (%).

Indices	C	S12	S20	S28	SEM	Treatment	L	C	Q
SFA	50.43 ^a^	45.40 ^b^	48.54 ^ab^	52.82 ^a^	1.58	*	NS	NS	**
USFA	49.56 ^b^	54.61 ^a^	51.5 1^a^	47.17 ^b^	1.58	*	NS	NS	**
MUFA	44.26 ^ab^	49.11 ^a^	46.38 ^a^	41.00 ^b^	1.56	**	NS	NS	**
PUFA	5.42	5.58	5.19	6.35	0.57	NS	NS	NS	NS
n3	0.19 ^b^	0.33 ^a^	0.26 ^ab^	0.21 ^b^	0.03	*	NS	NS	**
n6	4.14	4.40	3.91	4.78	0.47	NS	NS	NS	NS
SFA/USFA	1.03 ^ab^	0.84 ^b^	0.94 ^ab^	1.14 ^a^	0.06	*	NS	NS	***
n6/n3	21.75 ^a^	13.23 ^b^	15.37 ^b^	22.16 ^a^	1.06	***	NS	NS	***

C, 0% sunflower hulls (SFH); S12, 12% SFH; S20; 20% SFH; S28, 28% SFH; SFA, saturated fatty acid; USFA, unsaturated fatty acid; MUFA, monounsaturated fatty acid; PUFA, polyunsaturated fatty acid; and NS, no significant effect. * Significant effect (*p* < 0.05); ** significant effect (*p* < 0.01); and *** significant effect (*p* < 0.001). L: linear, C: cubic, and Q: Quadratic. ^a,b^ Means within a row with different superscript letters significantly differ.

## Data Availability

The data presented in this study are available on request from the corresponding author.
